# KymoTip: high‐throughput characterization of tip‐growth dynamics in plant cells

**DOI:** 10.1111/tpj.70691

**Published:** 2026-01-20

**Authors:** Zichen Kang, Yusuke Kimata, Tomonobu Nonoyama, Toru Ikeuchi, Kazuyuki Kuchitsu, Satoru Tsugawa, Minako Ueda

**Affiliations:** ^1^ Faculty of Systems Science and Technology Akita Prefectural University 84‐4 Yurihonjo Akita 015‐0055 Japan; ^2^ Graduate School of Life Sciences Tohoku University Sendai 980‐8578 Japan; ^3^ Department of Applied Biological Science Tokyo University of Science 2641 Yamazaki, Noda Chiba 278‐8510 Japan

**Keywords:** technical advance, KymoTip, cell tip detection, *Arabidopsis thaliana*, *Marchantia polymorpha*, centerline extraction, coordinate normalization, elongation direction, nuclear dynamics quantification, microtubule dynamics quantification

## Abstract

Live imaging data analysis often requires an objective, local, and accurate way of quantification of cell dynamics. In the research field of polarized tip‐growth, the cell fluctuations and/or fluctuations in tip position and growth direction hamper automated analyses of huge amounts of imaging sequences. The fluctuated nature in data makes it unclear how cell shape and growth are linked to intracellular events that could be the actual driving force of cell growth. To overcome these difficulties, we developed a powerful and user‐friendly tool called KymoTip with an available format. In this software, novel functions such as coordinate normalization, tip‐bottom detection, and signal kymograph were implemented. We confirmed that not only plasma membrane‐labeled fluorescent images, but also images such as bright‐field and cortical microtubule markers—so long as the cell contours can be identified—are amenable to KymoTip. Furthermore, by combining markers for cell contours with those that visualize intracellular structures, it becomes possible to quantitatively analyze various intracellular events, such as nuclear migration and calcium wave, in conjunction with cellular growth dynamics. Since KymoTip can be handled by non‐specialists, it is expected to promote understanding of what happens at the sub‐ and cellular level with high‐throughput outcomes.

## Graphical abstract

Fluctuations in live imaging data hamper analyses of tip‐growth dynamics. We developed KymoTip, a user‐friendly tool that uses coordinate normalization and centerline‐based tip detection to enable high‐throughput, quantitative analysis of both cell growth and its relationship with intracellular events.

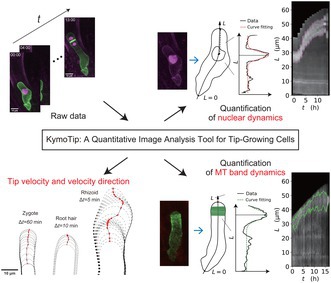

Faced with fluctuations in cell coordinates and cell tip positions, position correction of live imaging data and accurate detection of tip position are key challenges in plant developmental biology. We solved them with a powerful and user‐friendly tool, KymoTip, that can realize cell position correction, cell tip detection with cell centerline, and quantification of intracellular events.

## INTRODUCTION

Tip‐growth dynamics is an important research topic from the viewpoint of developmental biology. For example, an anisotropic elongation of plant zygote in *Arabidopsis thaliana* (Arabidopsis) was recently found to be the type of tip growth, indicating that tip growth may be essentially important for understanding the first recognition of the apical‐basal axis for the zygote in development (Kang et al., 2023). This finding facilitates obtaining different types of new live‐cell imaging because now it is possible to compare zygotes to well‐known tip‐growing cells such as pollen tubes and root hairs, so that huge amounts of data with high‐quality images and a well‐stored database including the wild type, mutants, and those with pharmaceutical treatment are ready to be analyzed (Hiromoto et al., 2023; Kimata et al., 2023; Matsumoto et al., 2021). These analyses revealed various polar dynamics, such as mitochondrial migration toward the growing cell (Kimata et al., 2020). The relationship between polarity dynamics and cell type function remains unclear. However, due to the lack of a simple method capable of simultaneously capturing the continuously deforming cell outline and the dynamics of intracellular structures and events, quantitative analysis of intracellular dynamics in relation to cell growth still relies on static images and/or manual observation.

The main difficulty that hampers the data analysis was the cell fluctuation due to the experimental observation with the seeds in the liquid medium so that it needs a certain coordinate normalization method at the analyzing stage to fix the zygote cell contours. This positional fluctuation made it disabled to observe intracellular dynamics in a spatially rearranged manner. For other examples, the root hairs in Arabidopsis and the rhizoid in the liverwort *Marchantia polymorpha* (Marchantia) exhibited tip growth to anchor the plant body (Otani et al., 2018; Shaw et al., 2000). The difficulty in this case was the fluctuation of the cell tip positions, which often required researchers to stay focused and detect the tip manually as a first attempt so that an objective detection of the cell tip may break down the barriers. In these examples, the quantification and characterization of cytoplasmic signals relative to the growing tip of cells have also been required. However, automated data analysis of tip positional detection is challenging because the cell tip positions are often fluctuating.

In recent years, quantitative approaches that promote the analysis of the inner dynamics of plants and their interactions have profoundly influenced the field of plant biology (Autran et al., 2021; Hamant, 2024). In recent years, with the advancement of live‐cell imaging techniques, the demand for quantitative image analysis that is capable of automated batch processing has been steadily increasing. For instance, PlantVis automatically analyzed the spatiotemporal behavior of root growth in Arabidopsis (Roberts et al., 2010; Wuyts et al., 2011). Similarly, RootflowRT quantified the growth rate of different types of root species (van der Weele et al., 2003), and KineRoot calculated growth rate without using characteristic markers for growth analysis (Basu et al., 2007). Recently, DIRTu was developed for quantifying root hair traits (Pietrzyk et al., 2025). These software programs achieved objective detection of growth rate. The growing region can be detected by GrowthTracer, where the displacement of any positions of root can be quantified using a certain filter (Iwamoto et al., 2013). For extraction of the growth rate along the cell axis, KymoRod uses Voronoi tessellation to detect the cell centerline objectively (Bastien et al., 2016). Admitting that all these existing tools can quantify the growth rate or growth region of the plant organs, none of these techniques identify the cell tip due to the existence of cell fluctuations in position and in growth. Therefore, it has been required to develop an objective and accurate way of cell tip detection in plant cell biology.

In this study, we propose a novel user‐friendly tool KymoTip with objective and accurate quantifications based on coordinate normalization. Firstly, we demonstrated a flow map of data analysis and concrete output of our analysis. Next, we showed the sufficiency of our method for quantitative analysis of cell growth dynamics using three examples: Arabidopsis zygotes, root hairs, and Marchantia rhizoids. Finally, we show the simultaneous analyses of cell growth dynamics and various intracellular events, and discuss how the objectivity and accuracy of tip growth are important for plant cell biology.

## RESULTS AND DISCUSSION

### Algorithm and series of output by KymoTip

The algorithm to evaluate the tip‐growth dynamics consists of three main steps: (1) Binarization/Segmentation to get the cell contours and their centerlines over time, (2) Coordinate normalization if necessary, and (3) Tip‐bottom detection by extending the Voronoi tessellation. We note that this tool is user‐friendly because of its accessibility to the code without complex hardware setups.

**Figure 1:**
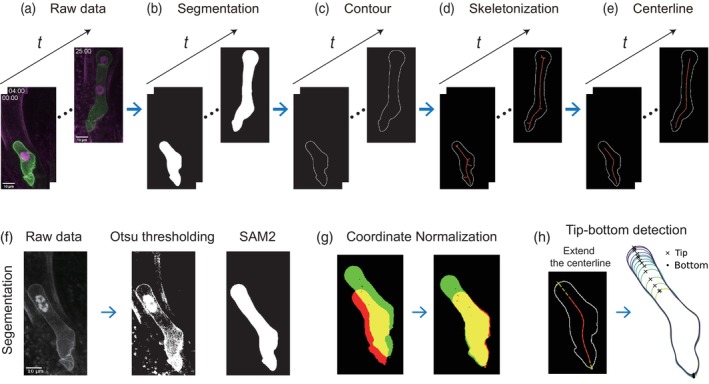
Workflow of KymoTip. (a) Raw data. Scale bars, 10 μm. (b) Binarization/Segmentation. (c) Cell contours. (d) Skeletonization using Voronoi tessellation. (e) Cell centerline. (f) Comparison between Otsu thresholding and SAM2. (g) Coordinate normalization of two successive images. (h) Tip‐bottom detection by extending the centerline to the cell edge.

At first, we obtained the cell segmentation and cell contours to get cell centerline from the original raw data, that is, time‐lapse images of Arabidopsis zygotes expressing green and red fluorescent markers labeling the plasma membrane and nucleus, respectively (Figure 1a–c). In this process, obtaining precise cell contours is critical, because the subsequent skeletonization based on Voronoi tessellation is highly sensitive to noise in cell contours and often results in small branches that correspond to the noise on the cell contour. Therefore, we applied a method of segmentation based on SAM2 (Segment Anything Model 2) which enables a more accurate segmentation rather than a canonical method like Otsu (Ravi et al., 2024, see Experimental Procedures, Figure 1f). This deep‐learning‐based method enables robust and highly precise segmentation, ensuring smooth cell contours necessary for accurate centerline detection. Specifically, we utilized the *sam2_hiera_large* model checkpoint. For the initialization of the segmentation in the first frame, we provided prompts (e.g. positive clicks or bounding boxes) targeting the cell to the model. The generated masks are then propagated to the subsequent frames using the video predictor module of SAM2, which maintained the consistency of the tracked cell. In contrast to traditional machine learning segmentation methods like *ilastik* (Berg et al., 2019), SAM2 allows KymoTip to robustly segment various plant cell shapes immediately, without the need to train a classifier for each specific dataset. We then obtained a skeletonization profile using Voronoi tessellation of the point cloud on the cell contour (Figure 1d, see also Bastien et al., 2016). In order to extract the primary centerline representing the cell axis, we removed the small branching by detecting the intersections of skeletons and obtained the centerline that does not reach the contour (Figure 1e). In these processes, we applied coordinate normalization detailed in the experimental procedures (Figure 1g) and detected the cell tip and bottom by extending the centerline (Figure 1h).

### Applicability of KymoTip to various tip‐growing cells

**Figure 2:**
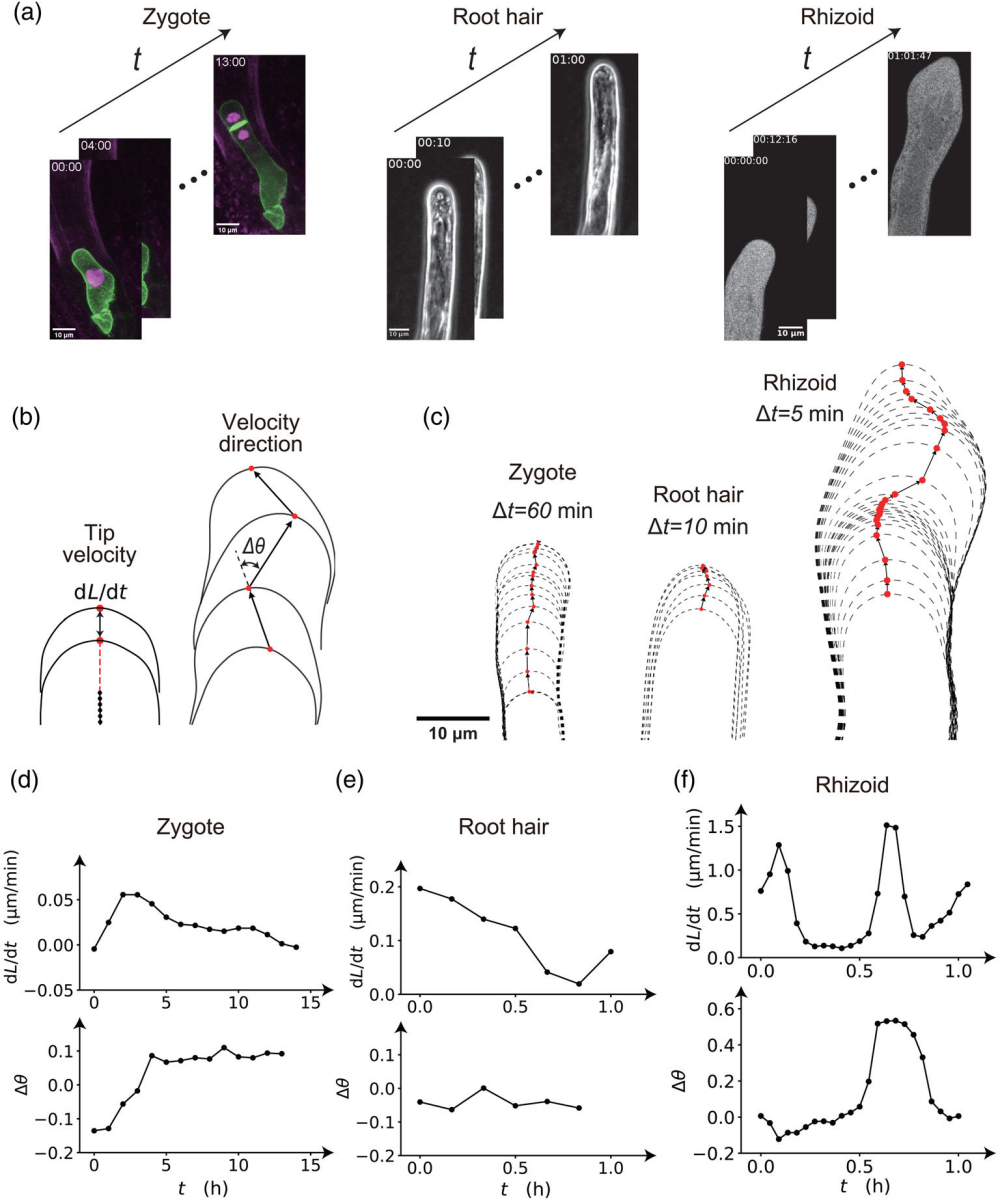
Versatility of the KymoTip of detections of the tip‐bottom, tip velocity and velocity direction. (a) Raw data of the Arabidopsis zygote, Arabidopsis root hair, and Marchantia rhizoid. (b) Schematic illustrations of the tip velocity d*L*/d*t* and the velocity direction Δ*θ* by tracking the cell tip over time. (c) Examples of tip detections of the Arabidopsis zygote, Arabidopsis root hair, and Marchantia rhizoid. Δ*t* denotes the time interval of the image sequences. (d–f) Quantitative results of tip velocity and velocity direction of the Arabidopsis zygote (d), Arabidopsis root hair (e), and Marchantia rhizoid (f).

We propose new methods of detecting the cell tip position, velocity, and direction. We put three examples of tip‐detected results for zygote, root hair, and rhizoid (Figure 2a). Using tip positions, we could calculate the tip velocity d*L*/d*t* with the cell length *L* and the velocity direction Δ*θ* as schematically illustrated in Figure 2(b). These parameters are derived from the displacement between consecutive time points; a minimum of two frames is required for the analysis. Using tip positions of zygote (Figure 2c left), we obtained that the tip velocity was maximally 0.05 μm min^−1^ and the velocity direction indicates that the tip first faces to the left and gradually changed the direction to the right in this example (Figure 2d). In the case of root hair of time‐lapse bright‐field images (Figure 2c center), the cell centerline and tip detection were performed without the coordinate normalization. We note that when the cell bottom was fixed experimentally, the coordinate normalization is not necessary. Then, we obtained that the tip velocity was maximally 0.2 μm min^−1^, roughly estimated by 4 times faster than zygote, in which the velocity direction is not significantly changed (Figure 2e). From raw data of *mCherry* fluorescence in the rhizoid (method), it shows bumpy cell shapes over time (Figure 2c right). The maximal tip velocity was 0.6 μm min^−1^, which was roughly three times faster than root hairs (Figure 2f). More interestingly, when the tip velocity becomes higher, the velocity direction is also changed synchronously. Since rhizoids typically elongate while bending to respond to environmental cues, the observed synchronization of velocity and angle likely reflects this intrinsic growth behavior. Although the biological mechanisms driving this synchronization, including molecular, genetic, and ionic factors, are still not fully understood, this tool will be a powerful platform when the behavior of organelles and molecular dynamics within cells become clearer.

To validate the measurement accuracy of KymoTip, we analyzed previously published time‐lapse data of Arabidopsis root hairs (movie S3 from Singh et al., 2021). The quantification results of tip displacement and nuclear movement by KymoTip were consistent with the reported data (Figure S1).

### Quantification of cytoplasmic signals

We further investigated whether KymoTip enables simultaneous tracking of intracellular events in relation to changes in cell morphology. For this purpose, we examined the spatiotemporal dynamics of nuclear migration toward the apical cell tip and the constant accumulation of a microtubule band in the subapical region of the zygote (Kang et al., 2024; Kimata et al., 2016).

**Figure 3:**
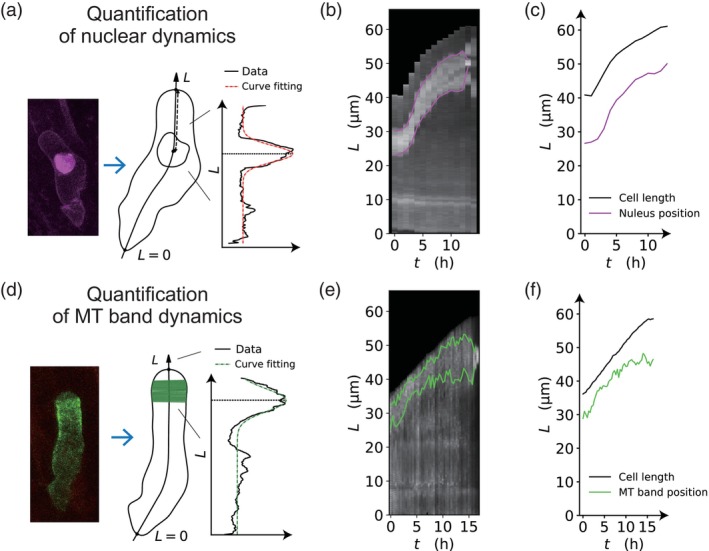
Characterization of intracellular dynamics in the Arabidopsis zygote. (a) Quantification of nuclear dynamics in zygote by fitting the curve of the nucleus fluorescence intensity (Kang et al., 2024). (b) Kymograph of nucleus fluorescence based on coordinate normalization in zygote. Magenta lines show upper and lower ends of the nucleus. (c) Quantitative comparison of cell length and nucleus position in zygote. (d) Quantification of microtubule band by Gaussian fitting of the microtubule fluorescence intensity in zygote (Kang et al., 2024, BioRxiv). (e) Kymograph of microtubule band based on coordinate normalization in zygote. Green lines show upper and lower ends of the microtubule band. (f) Quantitative comparison of cell length and microtubule band position.

First, the nuclear position was determined using dual‐color imaging of the cell outline and nuclear markers (as in Figure 1a). We applied KymoTip to extract the cell centerline from the cell contour (Figure 1), and fitted the fluorescence profile of the nuclear signal using a truncated Gaussian distribution (Figure 3a). Based on these data, a kymograph was generated (Figure 3b), allowing simultaneous quantification of both cell growth and nuclear movement (Figure 3c). These results demonstrate that various intracellular dynamics can be precisely quantified by converting them into a well‐defined coordinate system along the cell centerline, enabled by co‐visualization with a cell contour marker. Furthermore, when using live imaging data of microtubule markers, the cell shape was inherently visualized by the marker itself (Figure 3d). Thus, even without an additional cell contour marker, KymoTip successfully enabled the tracking of microtubule position in the cell coordinate system (Figure 3d–f). In this context, the constant microtubule band in the subapical region was reliably detected using truncated Gaussian fitting (Figure 3e,f). These features highlight the significant advantages of KymoTip.

Importantly, the intracellular quantification module of KymoTip is versatile. It is applicable not only to the analysis of fluorescent probes or dyes (in vivo) but also to transgenic lines overexpressing fluorescent proteins.

### Summary and future perspectives

In summary, we developed a powerful tool KymoTip to assess tip‐growth dynamics, allowing improved segmentation with SAM2, coordinate normalization, tip detection by centerline extraction, quantification of growth direction, and an evaluation of the spatial distribution of cytoplasmic signals in plant cells. The first highlight was that an improved cell contour segmentation of SAM2 resulted in more accurate precision of coordinate normalization, enabling the fixation of cell fluctuations. The second highlight was that an idea of extension of the centerline leads to an accurate detection of the cell tip, enabling the quantification of growth direction and spatial cytoplasmic signals. The method is available on the internet, which prompts understanding of various tip‐growth dynamics for ubiquitous users.

For future perspectives, three novel properties will be available to analyze in tip‐growing dynamics: cell contours with coordinate normalization, tip positions, and its growth directions. With respect to the normalized cell contours, it allows one to clearly see the cytoplasmic fluorescence being freed from visual fluctuations of cell position. In addition, a spatiotemporal curvature change of cell shape can be traceable in a well‐rearranged manner. With respect to tip positions, a dream of visualizing the cytoplasmic signals centering from the cell tip comes true. Furthermore, a relative distance of cytoskeleton signals such as microtubule band to the cell tip can be quantified, enabling one to think of its biological meaning during tip growth. With respect to tip‐growth directions, it may be possible to see correlations between the calcium waves and growth direction, and it may also be able to understand the relationship between growth rate and growth direction. Importantly, the KymoTip method is not limited to a specific plant species or cell type. When combined with our recently developed technique that enables the visualization of the living zygotes across a wide range of plant species using only fluorescent staining (Hanaki et al., 2025), KymoTip offers a general framework for high‐resolution and quantitative analysis of cellular dynamics beyond conventional model organisms. Thus, KymoTip definitely opens a new avenue for understanding multi‐faceted aspects of tip‐growth and cellular behavior across plant lineages. While the current version of KymoTip focuses on the growth dynamics of single‐tip cells, the centerline‐based approach is extensible to more complex shapes. In the case of multiple protrusion growth, calculating multiple centerlines makes it possible to analyze multiple tips simultaneously. Extending KymoTip to handle such multiple‐tip quantification remains an important task for future development.

## EXPERIMENTAL PROCEDURES

### Image acquisition and pre‐treatment

Live imaging data of the plasma membrane and nucleus in Arabidopsis zygotes were obtained using an *in vitro* ovule cultivation method combined with two‐photon excitation microscopy, and were used in a previous study (Kang et al., 2023; Ueda et al., 2020). Time‐lapse imaging data of zygotic microtubules and root hair morphology (Movie S1) have also been previously reported (Kang et al., 2023, 2024).

For imaging of *M. polymorpha* rhizoids, the wild type (Tak‐1) plants expressing mCherry were grown as reported previously (Watanabe et al., 2024). Gemmae were floated in 10 mL of MES buffer (0.5 mM CaCl_2_, 50 mM sorbitol, 0.25% low melting point agar, pH 5.7) and incubated for more than 16 h under continuous white light. For imaging, several gemmae were transferred into a drop of 10 mM MES buffer placed at the center of a coverslip bordered on all sides with vinyl tape, then sealed with a second coverslip to create a shallow chamber, where the gemmae were immobilized between the two coverslips (Movie S2). Time‐lapse imaging of mCherry fluorescence (excited by 577 nm laser and detected 561–628 nm) was performed by a confocal laser scanning microscope LSM900 (Carl Zeiss).

### Algorithms of the detections of contour and cell centerline

We used a deep‐learning‐based model SAM2 (Ravi et al., 2024) for segmentation of the images. In this process, we need to identify the cell as an object by pointing to a specific point inside the cell. Using the points of the resulting cell contour, we applied the Voronoi tessellation associated with *xy*‐coordinates of the cell contour to detect a skeletonization of the cell. Then, the centerline was obtained by cutting the small branches. We used *x* points near the end of the branch‐cut skeletonization and extrapolated them linearly. We can use Lowess for cell contours and/or the resulting centerline to smooth the curves.

### Coordinate normalization

We rearranged the cell contours using an image‐based coordinate normalization (Kang et al., 2023). We define $f_{\mathrm{ref}}\left(x,y\right)$ as the fluorescence intensity of the reference image at pixel coordinates $\left(x,y\right)$, and $f\left(x\left(\theta \right),y\left(\theta \right)\right)$ as the fluorescence intensity of the rotated image at the corresponding coordinates determined by the rotation angle θ. The variables u,v are the translational shift in x‐ and y‐directions, respectively. We then calculated a normalized correlation function written as,

$$\gamma \left(u,v,\theta \right)=\frac{\sum_{x,y}\left[f_{\mathrm{ref}}\left(x,y\right)-{\bar{f}}_{\mathrm{ref}}\right]\left[f\left(x\left(\theta \right)-u,y\left(\theta \right)-v\right)-\bar{f}\right]}{\sqrt{\sum_{x,y}{\left[f_{\mathrm{ref}}\left(x,y\right)-{\bar{f}}_{\mathrm{ref}}\right]}^{2}\cdot \sum_{x,y}{\left[f\left(x\left(\theta \right)-u,y\left(\theta \right)-v\right)-\bar{f}\right]}^{2}}}$$

Here, $\bar{\cdot }$ is the spatial average of ·. We note that the values of $f\left(x\left(\theta \right)-u,y\left(\theta \right)-v\right)$ are set to 0 in regions that fall outside the image boundaries after rotation and/or translation. We optimized the parameters u,v and θ to maximize the correlation function γ and thereby align the image sequences with the reference frame.

## Conflict of Interest

The authors have no conflict of interest.

## Supporting information


**Figure S1.** Quantification of cell growth and nuclear dynamics using KymoTip. The time‐lapse data were obtained from Movie S3 provided in the supplementary materials of Singh et al. (2021).

**Movie S1.** Cell growth dynamics of the Arabidopsis root hairs. Time‐lapse observation of the root hairs. Numbers indicate the time (h:min) from the first frame. Images were obtained at 10‐min intervals. Scale bar: 10 μm.

**Movie S2.** Cell growth dynamics of the Marchantia rhizoid. Time‐lapse observation of the rhizoid. Numbers indicate the time (h:min:sec) from the first frame. Images were obtained at 1.64‐sec intervals. Scale bar: 10 μm.

## Data Availability

The code for KymoTip is available on GitHub: https://github.com/blues0910/KymoTip and the code for SAM2 segmentation is available at https://github.com/YusukeKimata‐Moo/SAM2‐segmentation/. The raw data used in this paper are available on figshare: https://doi.org/10.6084/m9.figshare.30847580.
