# Supplementary material for: KymoTip: high‐throughput characterization of tip‐growth dynamics in plant cells
Source: Plant J. 2026 Jan 20;125(2):e70691. doi: 10.1111/tpj.70691 (PMC12818910; doi:10.1111/tpj.70691)
Supplement: Supplementary file 1 — Figure S1. Quantification of cell growth and nuclear dynamics using KymoTip. The time‐lapse data were obtained from Movie S3 provided in the supplementary materials of Singh et al. (2021). [file TPJ-125-0-s001.docx]

Supplementary material

Supplementary Figures

**
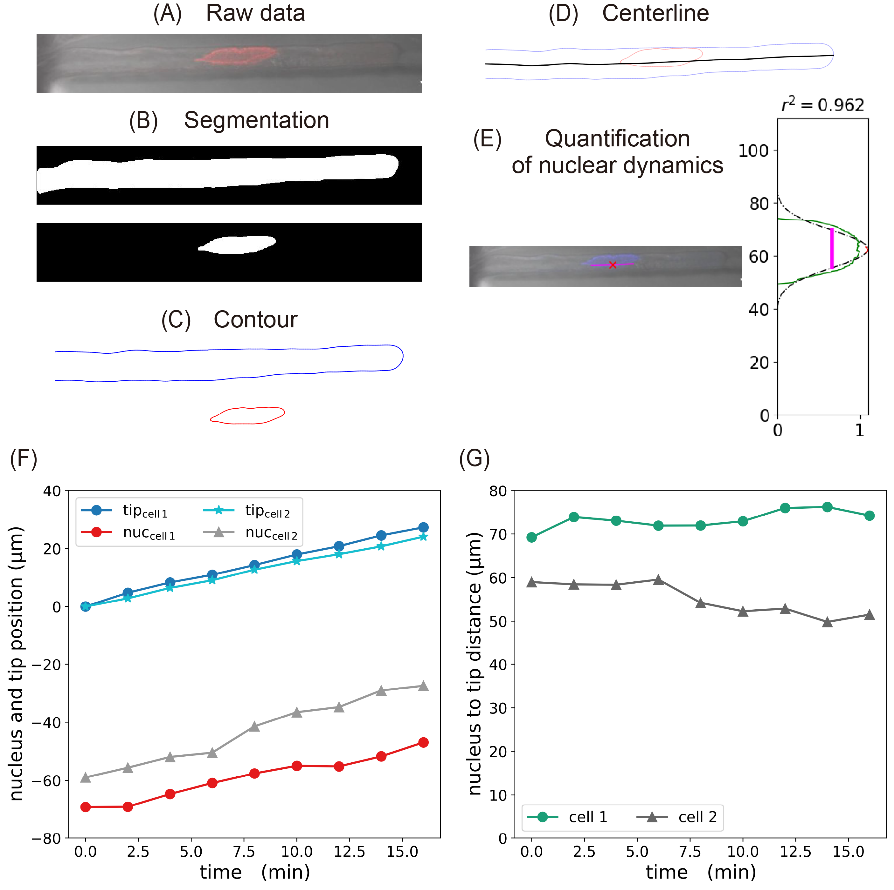
**

**Fig. S1.** Quantification of cell growth and nuclear dynamics using KymoTip. The time-lapse data were obtained from Movie S3 provided in the supplementary materials of Singh et al., 2021. (A) Raw data. (B) Segmentation results for the cell (upper) and nucleus (lower). (C) Extraction of cell and nuclear contours from the segmented images. (D) Computation of the cell centerline from cell contour. (E) Quantification of nuclear dynamics along the centerline. (F) Temporal changes in cell tip position and nuclear position for two cells. (G) Distance between the nucleus and the cell tip over time for cells.

Supplementary Movies

**Movie S1.** Cell growth dynamics of the Arabidopsis root hairs. Time-lapse observation of the root hairs. Numbers indicate the time (h:min) from the first frame. Images were obtained at 10-min intervals. Scale bar: 10 µm.

**Movie S2.** Cell growth dynamics of the Marchantia rhizoid. Time-lapse observation of the rhizoid. Numbers indicate the time (h:min:sec) from the first frame. Images were obtained at 1.64-sec intervals. Scale bar: 10 µm.
