# Supplementary figures and images for: KymoTip: high‐throughput characterization of tip‐growth dynamics in plant cells
Source: Plant J. 2026 Jan 20;125(2):e70691. doi: 10.1111/tpj.70691 (PMC12818910; doi:10.1111/tpj.70691)

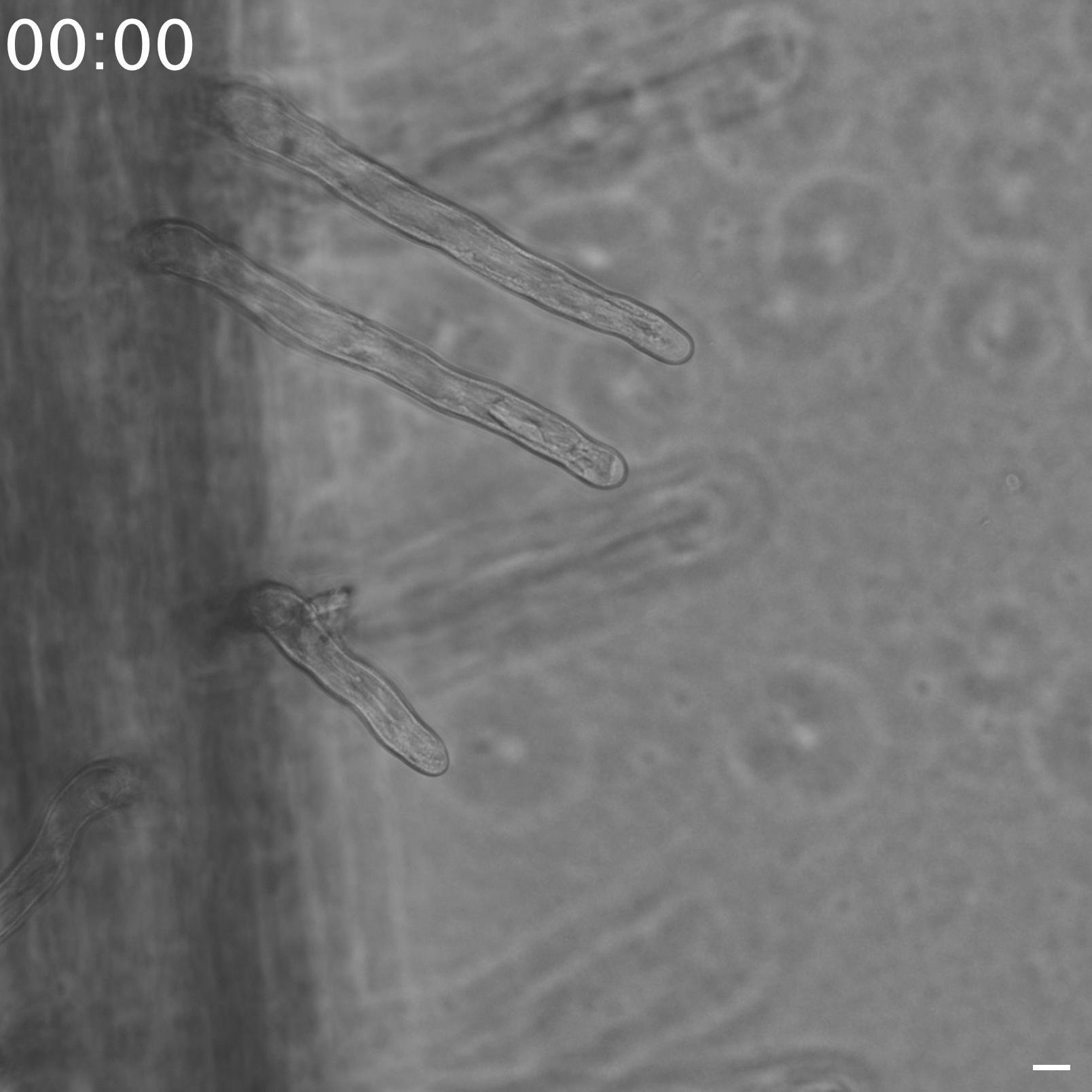

Supplement: Supplementary file 2 — Movie S1. Cell growth dynamics of the Arabidopsis root hairs. Time‐lapse observation of the root hairs. Numbers indicate the time (h:min) from the first frame. Images were obtained at 10‐min intervals. Scale bar: 10 μm. [file TPJ-125-0-s002.zip › tpj70691-sup-0002-MovieS1/MovieS1_thumbnail.jpg]

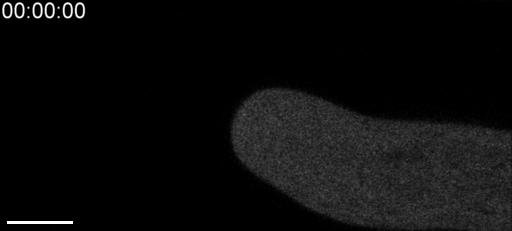

Supplement: Supplementary file 3 — Movie S2. Cell growth dynamics of the Marchantia rhizoid. Time‐lapse observation of the rhizoid. Numbers indicate the time (h:min:sec) from the first frame. Images were obtained at 1.64‐sec intervals. Scale bar: 10 μm. [file TPJ-125-0-s003.zip › tpj70691-sup-0003-MovieS2/MovieS2_thumbnail.jpg]
